# Del nido cardioplegia in adults: a retrospective observational study in comparison to modified St. Thomas cardioplegia in cardiac surgery

**DOI:** 10.1186/s13019-024-02683-1

**Published:** 2024-04-25

**Authors:** Sanjeeve Kumar Garg, Rajinder Singh Rawat, Dina Afifi Saad, Ihab Omar

**Affiliations:** 1 Cardiothoracic Surgery Department, Sultan Qaboos Hospital, Salalah, Oman; 2 Cardiothoracic Anesthesia Department, Sultan Qaboos Hospital, Salalah, Oman; 3 Cardiothoracic Surgery Department, Faculty of Medicine, Benha University, Benha, Egypt; 4 Cardiothoracic Surgery Department, Faculty of Medicine, Beni-Suef University, Beni Suef, Egypt

**Keywords:** Del Nido, Modified St Thomas, Cardioplegia, Cardiac surgery methods

## Abstract

**Background:**

St. Thomas cardioplegia is commonly administered to adults, yet repeated dosing at brief intervals is required. Del Nido’s cardioplegic solution provides a prolonged duration of safe myocardial arrest, yet it was primarily intended for pediatric cardiac surgery. Recently, there has been an increasing interest in using Del Nido’s in adults; this might be due to its ease of administration and extended re-dosing intervals. This study contrasted Del Nido’s to modified St. Thomas cardioplegia in adults.

**Methods:**

This study was conducted on 200 patients. Troponin-T was the primary outcome within the first 24 and 48 h post-surgery. Cardiopulmonary bypass time, cross-clamp time, intraoperative use of inotropic support, defibrillator and/or intra-aortic balloon were the secondary outcomes of the study.

**Results:**

There was a significant reduction in post-operative Troponin-T levels in the first 24 and 48 h within Del Nido’s group compared to the modified St. Thomas group. The cross-clamp and cardiopulmonary bypass times were also found to be lower within Del Nido’s group.

**Conclusion:**

This study has demonstrated a significant reduction in early postoperative Troponin-T levels as well as operative times favoring Del Nido’s in adults.

## Background

Cardioplegia is a technique that temporarily halts the heart, safeguards the myocardium, and maintains normal heart function in patients undergoing cardiac surgery. The cardioplegic solutions contain crystalloid or blood-based substances, electrolytes, energy substrates, and pharmaceutical additives [1–3]. Del Nido (DN) cardioplegia is a well-known cardioplegia solution developed by Dr. Pedro Del Nido in 1990, which consisted of a blood-supplemented crystalloid solution with low potassium and calcium and high sodium and magnesium concentrations [4–6]. This cardioplegia formulation is well-known in the medical industry due to its extended duration of cardiac protection, ease of administration, and decreased re-dosing frequency. Yet, few studies have been conducted on its use in adults [3, 4, 7–17]. However, due to extended re-dosing intervals achieved with DN and advances in scientific research, it has become appealing for surgeons to extend its use in adults, where it has been reported to provide satisfactory outcomes [7, 10, 11, 18, 19].

It is reported that DN’s can be used safely in long-duration adult cardiac surgeries and a single dose with better intraoperative and immediate post-operative outcomes as compared to St Thomas’s solution [9]. Mishra et al. [12] found that the two solutions produced comparable postoperative mortality and substantial cardiovascular adverse events. Nevertheless, these findings are inconclusive; additional research is required to determine whether modified St. Thomas (MST) or DN’s cardioplegia is preferable. There is still considerable debate regarding the optimal cardioplegic solution which is to be used in adult cardiac surgery. Therefore, in this present study, we aimed to evaluate the relative benefits and risks of DN cardioplegia and MSTcardioplegia by contrasting the clinical outcomes of patients treated with both cardioplegias. Similarities and differences between the two cardioplegia options regarding clinical outcomes were observed pre-, intra and post-operatively.

## Methods

### Data collection

After obtaining the study and research committee approval, the research was conducted on 200 adult patients who were operated at Salalah Cardiac Centre -Sultanate of Oman at time frame between June 2016 and December 2021. Data were retrospectively analysed from the computer database, and patients were divided into two equal groups according to the type of administered cardioplegia. Del Nido (DN group, *n* = 100) and Modified St. Thomas (MST group, *n* = 100). Patients included in the study were from both genders (male and female), aged 20–80 years old. They had their first median sternotomy for elective coronary artery bypass grafting (CABG), valve surgery or both.

In contrast, patients with a previous history of cardiac surgery, unstable angina, elevated pre-operative troponin-T (> 0.04ng/ml), low ejection fraction (EF) of the left ventricle (< 30%), emergency cases, patients requiring pre-operative chemical or mechanical support and those with elevated Society of Thoracic Surgery Risk Score (STS > 4) were excluded from the study. The decision of whether to administer DN or modified St.Thomas cardioplegia was due to differing surgeons’ preferences. One group of surgeons preferred using DN, while the other preferred using modified St.Thomas cardioplegia for their patients.

### Demographic data and clinical outcome

Demographic details comprised the patient’s age, sex, and body surface area (BSA). Whereas the clinical outcomes were EF, cardiopulmonary bypass (CPB) time, cross-clamp (XC) time, DC shock requirement, intra-operative inotropic support, use of intra-aortic balloon (IAB) and Troponin-T levels at 24 and 48 h after transfer to the intensive care unit (ICU) were also evaluated **(**Tables 1 and 2**).**

**Table 1:** Comparison between demographic and preoperative clinical outcomes of DN and MST group cardioplegia

Outcomes	DN group (*n* = 100)	MST group (*n* = 100)	P-value*
Age (years)	54 ± 1	51 ± 1	0.090
Gender (males, %)	84 ± 0.36	81 ± 0.39	0.577
BSA (m²)	1.8 ± 0.0	1.8 ± 0.0	0.812
Pre-Op EF (%)	52.7 ± 0.9	54.6 ± 0.95	0.151
STS risk score	1.3 ± 1.1	1.4 ± 1.0	0.859

*Values are mean ± standard deviation (SD) of samples. The significant difference is at *P* < 0.05

DN: Del Nido solution

MST: Modified St.Thomas solution

BSA: Body Surface Area

EF: Ejection Factor

STS: Society of Thoracic Surgeons Risk Score

**Table 2:** Comparison between intra-operative clinical outcomes of DN and MST cardioplegia

Outcomes	DN group (*n* = 100)	MST group (*n* = 100)	P-value*
XC time (minutes)	90 ± 4	105 ± 4	0.006
CPB time (minutes)	137 ± 6	177 ± 7	0.001
DC shock (%)	14.00 ± 0.33	33.00 ± 0.47	0.001
Inotropes and/or pressors not required (%)	10.00 ± 0.63	9.00 ± 0.61	0.256
Low (%) (1–2 inotropes and/or pressors < 50 ng/kg/min)	42.00 ± 0.41	41.00 ± 0.41	0.256
Moderate (%) (1–2 inotropes and/or pressors ranging from 50–100ng/kg/min each)	29.00 ± 0.50	22.00 ± 0.55	0.256
High (%) (1–2 inotropes and/or pressors > 100ng/kg/min each) or 3 inotropes and/or pressors.	19.00 ± 0.57	28.00 ± 0.50	0.256
IAB (%)	9.00 ± 0.56	7.00 ± 0.28	0.602

*Values are mean ± standard deviation (SD) of samples. The significant difference is at *P* < 0.05

DN: Del Nido solution

MST: Modified St.Thomas solution

XC: Cross Clamp

CPB: Cardiopulmonary bypass

DC: Direct Current

IAB: Intra-Aortic Balloon

### Operative details and cardioplegia administration

All patients were operated by using standard general anaesthesia protocol as per the institute’s standard of care. Consent for routine surgical procedures was obtained from all study participants. Median sternotomy approach, cardiopulmonary bypass, and mild hypothermia (30–32 °C) were the standard for all study participants.

Ready-made St. Thomas cardioplegia ampoules (Martindale Pharma) were obtained from within the facility and were used in MST group. The ampules were composed of 20 ml solution containing potassium chloride (60 mg/ml, 1.193 g), magnesium chloride hexahydrate (163 mg/ml, 3.253 g) and procaine hydrochloride (14 mg/ml, 272.8 mg), which were diluted over 200 ml ringers’ and mixed to 800 ml patient’s blood in a ratio of 1 unit of crystalloid to 4 units of blood. A separate pump head with a cooling coil was used to cool the mixture down to 8 °C and pump the plegia into the aortic root. On the other hand, the DN plegia was formulated manually by mixing the constitutes (Table 3) in their appropriate doses over 800 ml of ringers and adding them to 200 ml of the patient’s blood in a ratio of 4 units of crystalloid to 1 unit of blood, which was then delivered to the aortic root at 8 °C, as previously mentioned with MST. Both groups received an induction of freshly prepared ante-grade cardioplegia (dose 20 ml/kg, at temperature of 8 °C). Repeated doses of 10 ml/kg were provided every 25 min for MST group and every 90 min for DN group.

**Table 3:** DN cardioplegia formulation

DN composition	Dosage (mg/ml)	Dosage (g)
Mannitol	16.3	3.26
Magnesium sulfate	4	2
Sodium bicarbonate	13	1.09
Lidocaine	13	0.13
Potassium chloride	13	0.96

### Statistical analysis

Statistical analysis was performed using the statistical package software IBM-SPSS version 24 for analyzing the results. The Chi-square or Fisher’s exact test was used to study the associations among qualitative variables. The Shapiro-Wilk test was used to examine the distribution of the quantitative data. We used an unpaired Student’s t-test to compare the means. Duplicate samples were used to assess paired t-tests for comparing pre-and post-operative clinical outcomes from the same group with *P* < 0.05 as the limit of statistical significance.

## Results

### Pre-operative characteristics

The pre-operative risk score and baseline characteristics are represented in Tables 1 and 4. According to Table 1, the average age in the DN group was 54 ± 1 years old, while patients in the MST group, on average, were 51 ± 1 years of age. However, the *P*-value of 0.090, as indicated in the table, showed that the age difference between the two groups was not statistically significant. Moreover, males constituted 84% of patients in DN group and 81% in the MST group. Moreover, the average BSA was 1.8m^2^ in both group. Patients within DN had a preoperative left ventricular EF of 52.7% compared to a preoperative EF of 54.6% in MST group. Similarly, a mean STS risk score of 1.3 was characterized in the DN group, and an average score of 1.4 was for MST group patients. In conclusion, the represented *P*-values in the variables mentioned above between the groups (DN and MST) were insignificantly different.

**Table 4:** Comparison between the distribution of DN and MST group procedures

Outcomes	DN group (*n* = 100)		MST group (*n* = 100)	P-value*
Isolated CABG (%)		69	68	0.571
CABG + Valve (s) (%)		7	4	0.467
Isolated Valve (s) (%)		24	28	0.564

*The significant difference is at *P* < 0.05

DN: Del Nido solution

MST: Modified St.Thomas solution

CABG: Coronary Artery Bypass Grafting

Table 4 presented a comparison of the distribution of patients according to the type of surgery between the DN group and the MST group. The table included various variables representing different types of surgeries performed, the number of patients in each group who underwent those surgeries, and the *P*-values indicating the statistical significance, if any, observed between the two groups. 69% of patients in DN group underwent isolated CABG surgery, while 68% underwent isolated CABG surgery in the MST group. 7% of patients in DN group underwent combined CABG and valve surgery, and 4% in MST group underwent combined CABG and valve surgery. Moreover, 24% of patients in DN group underwent isolated valve surgery, whereas 28% underwent isolated valve surgery in MST group. Therefore, based on the information in the table, there is no statistically significant difference in the distribution of patients undergoing isolated CABG or isolated valve surgery between the DN and MST groups.

### Intra-operative data

The intra-operative characteristics of patients in DN and MST groups, along with their respective *P*-values, are presented in Table 2. According to the table, the time the patient was held in a XC during surgery for the DN group was 90.4 min, whereas for the MST group, it was 105.4 min. Indicating a significant reduction in XC time within the DN group. The number of minutes where the CPB was in effect during surgery was significantly reduced within the DN group to 137.6 min, compared to 177.7 min within MST group. DC cardioversion requirement was lower for DN groups (14%) than MST group (33%). However, the need for intra-operative inotropic or pressor support and IAB showed no significant differences between both groups.

Ten patients in the DN group and nine patients in the MST group did not need inotropes and/or pressors. At the same time, 42 patients in the DN group and 41 patients in the MST required low inotropes and/or pressors. Further, 29 patients from DN group and 22 patients from MST group went for moderate inotropes and/or pressors. Around 19 patients from DN group and 28 patients from MST group required high doses of inotropes and/or pressors, which all indicated no significant differences. Lastly, according to the results, 9% of the patients in the DN group needed IAB, and 7% in the MST group also needed an IAB. The need for an IAB does not differ significantly between the two groups. As a result, the data showed no statistical significance between the DN and MST groups regarding inotropic support or the necessity for an IAB pump.

### Post-operative data

Post-operative characteristics to compare DN group and the modified MST group were early Troponin-T levels and post-operative EF (%) represented in Table 5. The mean 24-hour (ng/ml) troponin concentration in the DN and MST groups was 1.44ng/ml and 2.54ng/ml, respectively. At 24 h after surgery, troponin levels were significantly lower in the DN group than in the MST group. Whereas troponin levels observed 48 h after surgery were significantly lower in the DN group compared to the MST group, which were recorded as 0.84ng/ml in DN groups and 1.42ng/ml in the MST group. However, there was no statistically significant change in EF between both groups following surgery, as the mean post-operative EF (%) for the DN group was 49.53% and for the MST group was 49.09%.

**Table 5:** Comparison between postoperative clinical outcomes of DN and MST groups

Outcomes	DN group (*n* = 100)	MST group (*n* = 100)	P-value*
Troponin 24 h (ng/ml)	1.44 ± 0.98	2.54 ± 2.37	0.001
Troponin 48 h (ng/ml)	0.84 ± 0.61	1.42 ± 1.59	0.001
Post-operative EF (%)	49.53 ± 9.56	49.09 ± 10.92	0.762

*Values are mean ± standard deviation (SD) of samples. The significant difference is at *P* < 0.05

DN: Del Nido solution

MST: Modified St.Thomas solution

EF: Ejection Fraction

Our study showed no intraoperative or early postoperative mortality; early post-operative liver derangement was witnessed in two patients, which resolved within a few days; early postoperative renal dysfunction was witnessed in three patients which were known to have preoperative chronic kidney disease and required postoperative dialysis.

## Discussion

The primary outcome parameter in this study was Troponin-T, which was traced at first 24 and then 48 h from the patient’s transfer to the ICU. Compared to troponin I, troponin T was robust and resistant to degradation, making troponin T level measurement less challenging, especially when dealing with a larger sample size and longer duration [20]. Moreover, previous studies supported using troponin T as a clinical outcome in various cardioplegic studies [6, 13, 21]. According to the National Academy of Clinical Biochemistry Medicine Practice Guidelines, peak cardiac troponin levels occurred approximately 18 h after the onset of symptoms [22–24]; thus, 24 h were sufficient for tracing peak troponin levels. The present study demonstrated a statistically significant reduction in Troponin-T values within the DN group compared to MST group. Although previous studies have shown reduced troponin levels in patients receiving DN cardioplegia, they have failed to show statistical significance, possibly due to insufficient sample standardization in terms of time intervals and the number of experiments [7, 10, 14].

Moreover, significantly shorter XC and CPB times were observed with DN group, which was consistent with earlier studies [21]. However, it’s not possible to determine from this study whether that was attributed to the streamlined surgical flow achieved with DN or simply a coincidence of shorter XC and CPB times. The DN group showed a significant reduction in intraoperative DC requirement, which was in accordance with studies by Mishra et al. [12] and Li et al. [16]. It might be attributed to the shorter ischemic times achieved with DN as well as the membrane stabilizing effect of lidocaine which increases sodium channel blockage and minimize the potential for sodium window current, together with its magnesium content acting as a calcium antagonist, protecting the myocardium from high intracellular calcium [6]. Regarding cardioplegia dosing, our current study demonstrated a single-dose administration in 75% of patients in the DN group. Other centres have reported rates ranging between 65% and 83% [12, 15, 17]. A lower rate of 40% was reported by Smigla et al. [7], which can be attributed to their 45-minute re-dosing policy. Sharma et al. [9] have shown a significant reduction in immediate postoperative inotropic support. However, our study was focused on intra-operative inotropes and/or pressors as we lack precise computerized records for their use in postoperative ICU.

Nevertheless, our results showed no significant difference between DN and MST regarding intra-operative inotropic or pressor support. Postoperative EF (%) showed no significant difference between our study groups, which contradicted Mishra et al. [12], who showed significant improvement in EF (%) with patients receiving DN cardioplegia. However, our study has certain limitations that should be considered. Firstly, a significant limitation is its retrospective nature with its inherited drawbacks, meaning that the information was gathered from previous medical records, which may contain some errors. The sample size is limited to 100 patients in each group, which limits the reliability of the results. Patients having other types of cardiac procedures were not included in the study as it was limited to those who underwent their first median sternotomy for elective CABG and/or valve surgery which further limits the study’s findings to a selected group of patients. Moreover, it is confined to a single-centre and compares two groups of patients who had surgery with different groups of surgeons. Moreover, long-term clinical outcomes including mortality, morbidity and length of hospital stay were not determined.

## Conclusion

This study concluded that in the future, DN cardioplegia could be added to the clinical guidelines and protocols for adult cardiac surgery. Incorporating the findings of this study to support the use of DN solution in adults could minimize myocardial injury and enhance early postoperative outcomes. Moreover, findings of this study indicated that DN cardioplegia offers advantages in terms of lowering Troponin-T levels, CPB periods and shortening XC. Subsequent investigations should endeavour to rectify these constraints and explore the enduring ramifications of implementing DN’s remedy, including variables such as morbidity, mortality, and comprehensive heart performance. Nevertheless, further investigation is necessary, including extended periods of observation and more extensive experiments, in order to thoroughly clarify the extensive advantages and possible therapeutic ramifications of using DN’s technique in the context of heart surgery for adult patients.

## Data Availability

Data is available and will be provided at the editor’s request.

## Abbreviations

DN: Del Nido
ICU: Intensive care unit
MST: Modified St. Thomas
CABG: Coronary artery bypass grafting
STS: Surgery Risk Score
BSA: Body surface area
EF: Ejection fraction
CPB: Cardiopulmonary bypass
XC: Cross-clamp
IAB: Intra-aortic balloon
